# Analysis of the Lung Microbiome in the “Healthy” Smoker and in COPD

**DOI:** 10.1371/journal.pone.0016384

**Published:** 2011-02-22

**Authors:** John R. Erb-Downward, Deborah L. Thompson, Meilan K. Han, Christine M. Freeman, Lisa McCloskey, Lindsay A. Schmidt, Vincent B. Young, Galen B. Toews, Jeffrey L. Curtis, Baskaran Sundaram, Fernando J. Martinez, Gary B. Huffnagle

**Affiliations:** 1 University of Michigan, Ann Arbor, Michigan, United States of America; 2 Veterans Affairs Health System, Ann Arbor, Michigan, United States of America; Charité-University Medicine Berlin, Germany

## Abstract

Although culture-independent techniques have shown that the lungs are not sterile, little is known about the lung microbiome in chronic obstructive pulmonary disease (COPD). We used pyrosequencing of 16S amplicons to analyze the lung microbiome in two ways: first, using bronchoalveolar lavage (BAL) to sample the distal bronchi and air-spaces; and second, by examining multiple discrete tissue sites in the lungs of six subjects removed at the time of transplantation. We performed BAL on three never-smokers (NS) with normal spirometry, seven smokers with normal spirometry (“heathy smokers”, HS), and four subjects with COPD (CS). Bacterial 16 s sequences were found in all subjects, without significant quantitative differences between groups. Both taxonomy-based and taxonomy-independent approaches disclosed heterogeneity in the bacterial communities between HS subjects that was similar to that seen in healthy NS and two mild COPD patients. The moderate and severe COPD patients had very limited community diversity, which was also noted in 28% of the healthy subjects. Both approaches revealed extensive membership overlap between the bacterial communities of the three study groups. No genera were common within a group but unique across groups. Our data suggests the existence of a core pulmonary bacterial microbiome that includes *Pseudomonas, Streptococcus, Prevotella, Fusobacterium, Haemophilus, Veillonella, and Porphyromonas*. Most strikingly, there were significant micro-anatomic differences in bacterial communities within the same lung of subjects with advanced COPD. These studies are further demonstration of the pulmonary microbiome and highlight global and micro-anatomic changes in these bacterial communities in severe COPD patients.

## Introduction

Chronic obstructive pulmonary disease (COPD) is a progressive and potentially fatal lung disease that is projected to be responsible for the fifth largest burden of disease worldwide by 2020 [1], [2]. COPD is characterized by largely irreversible airflow limitation, mucus hypersecretion, small airway fibrosis, and destruction of the alveolar space (emphysema) [3]. In developed nations, the leading cause of COPD is tobacco smoke exposure, predominately direct, whereas in developing nations indoor air pollution from combustion of biomass fuel also contributes significantly [4]. Not all smokers develop COPD, but why some do is not currently known. Because no current treatments halt COPD progression, new insights into its pathogenesis are urgently needed.

From the initial description of COPD as a distinct clinical condition responsible for productive cough and shortness of breath in patients without tuberculosis [5], there has been considerable controversy about the role of lower respiratory tract bacteria in its pathogenesis. This is the case both for its prolonged early asymptomatic phase and, until recently, for the acute exacerbations that punctuate its later stages [6], which can induce accelerated and sustained loss of lung function [7]. In part, this controversy arose because classical, culture-based studies suggested that the lungs of healthy individuals were sterile [8], [9], [10], while the lungs of COPD patients were believed to be colonized. More recently, culture-independent microbiological techniques demonstrated that the lungs are not sterile during health and documented changes in the lung microbiome in several lung diseases [11], [12], [13], [14], [15]. Nevertheless, the role of the lung bacterial microbiome in COPD pathogenesis and progression remains undefined.

Our two objectives addressed two gaps in the understanding of the pulmonary microbiome as related to smoking and COPD. The first was to assess the lung microbiome in smokers with neither signs of disease nor decreased lung function (“healthy” smokers) through analysis of bronchoalveolar lavage (BAL) fluid, which samples a broad region of lungs, and compare this to healthy non-smokers. The second objective was to determine whether pulmonary microanatomic/microenvironmental disparities lead to differences in the structure of localized bacterial communities in COPD, through analysis of multiple sample sites from surgical explants. We analyzed both types of samples by massively parallel pyrosequencing of bacterial 16S amplicons, a technique that provides a culture-independent analysis of the resident pulmonary microbiome and offers a breadth of analysis not previously available for studies of pulmonary biology and disease.

## Methods and Materials

### Ethics Statement

All clinical investigations were conducted according to the principles expressed in the Declaration of Helsinki. The study protocol was approved by the institutional review boards of the University of Michigan Healthcare System and the Ann Arbor Veterans Affairs Healthcare System. All patients provided written informed consent. The institutional review boards have examined the protocols and certified that “The risks are reasonable in relation to benefits to subjects and the knowledge to be gained. The risks of the study have been minimized to the extent possible.”

### Subject Enrollment – Patient populations

Specimens were obtained from subjects enrolled in an observational study registered with ClinicalTrials.gov as NCT00281229. Bronchoalveolar lavage (BAL) samples (n = 14) came from volunteers who underwent research bronchoscopy at the VA Ann Arbor Healthcare System. Surgical specimens (n = 8) were obtained from six patients undergoing clinically-indicated lung transplantation for COPD at the University of Michigan Health Care System. All subjects underwent pre-procedure spirometry, PA and lateral chest radiogram, electrocardiogram, complete blood count and automated chemistry analysis, prospectively collected medication history, and clinical evaluation; surgical participants also underwent computerized tomography (CT) of the chest and full pulmonary function testing. We excluded subjects who had mental incompetence or active psychiatric illness precluding informed consent; asthma as primary clinical pulmonary diagnosis; cystic fibrosis, clinically significant bronchiectasis or other inflammatory or fibrotic lung diseases; and those taking prednisone >20 mg daily.

Spirometry was expressed as a function of appropriate predicted equations for the included population [16], [17]. We categorized subjects using the spirometric classification of the Global Initiative for Chronic Obstructive Lung Disease (GOLD) [18]. For the BAL cohort analyses, subjects were segregated into three groups: healthy smokers (HS) exhibiting no evidence of underlying lung disease, post-bronchodilator FEV1/FVC>0.70 and FEV1%>80% predicted; never smokers (NS) having no smoking history or evidence of lung disease, post-bronchodilator FEV1/FVC>0.70 and FEV1%>80% predicted; COPD subjects (CS) having a post-bronchodilator FEV1/FVC<0.70 and FEV1%<80% predicted.

### Samples

Volunteer subjects underwent fiberoptic bronchoscopy under moderate conscious sedation according to published guidelines [19] using nebulized and instilled lidocaine, intravenous fentanyl, midazolam, and in some cases, diphenhydramine. Initially, we performed bronchoscopy via the nostril, which was anesthetized using viscous lidocaine (2%), but for the last 11 procedures, bronchoscopy was performed via the mouth to minimize contamination. The bronchoscope was successively wedged into a single subsegment of the right middle lobe and the lingula, each of which were lavaged with a total of 120 ml normal saline, heated to body temperature, which was removed using manual suction on a 30 ml syringe. The total recovered volume was pooled and transported to the laboratory immediately where 15 ml of unprocessed BAL fluid was reserved for microbiome analysis, before the remainder was processed for other research studies. BALs were divided evenly into 2 ml Eppendorf tubes, spun at 13,000 rpm for 30 minutes at 4°C in a microcentrifuge, the supernatants discarded, the pellets snap frozen in liquid nitrogen, and stored at -80°C.

Lung explants were obtained immediately following removal and were sterilely dissected in the hospital pathology lab in a laminar flow hood, using appropriate biosafety precautions. The lung explant was cut either sagittally or coronally to expose the airways for sampling. Airways were isolated starting proximally, and 1 cm^2^ tissue samples were sequentially collected from the segmental and distal portions of the upper, middle, and lower lobes, and processed separately. The anatomic location of sampling was carefully recorded to allow radiographic correlation with airway structure.

### Computed tomography

High resolution computed tomography (HRCT) images were acquired using 64-row detector CT scanners (GEMedical Imaging, Wisconsin, USA). After initial anterior-posterior and lateral planning images, supine end-inhalation axial images were obtained in a volumetric fashion. No intravenous contrast material was administered. The images obtained were 1.25 mm thick images obtained at every 1.25 mm interval with a pitch of1.375∶1, using 120 kvp, variable tube currents (ranged between 80-375 mA based on body habitus), 0.5-0.6 s tube rotation speed and with a noise index of 22. These images were reconstructed using bone algorithm and viewed using routine lung window settings (window width of 1300 HU and window level of -600 HU).

### DNA Isolation

To extract DNA from the BAL samples, tubes containing sample pellets (corresponding to 5 ml BAL) were suspended in a total of 500 µl Bacterial Lysis Buffer (BLB) (Roche Diagnostics), then transferred to 2 ml bead beating tubes (Mo Bio). Samples were homogenized in a Mini Bead-Beater 16 (Biospec) for 1 minute followed by centrifugation for 1 minute at 13,000 rpm in a fixed angle microcentrifuge (Microfuge 18, Beckman Coulter, Indianapolis, IN). Subsequently, 40 µl of proteinase K was added to each sample, which was then incubated for 10 minutes at 65°, followed by a second bead-beating for 1 minute, then centrifugation at full speed for 1 minute. Finally, tubes were incubated for 10 minutes at 95°C. DNA was harvested from these lysates using the MagNA Pure Compact system and Nucleic Acid Isolation Kit I (Roche, Indianapolis, IN). DNA concentration was quantified using the NanoDrop® ND-1000 Spectrophotometer (Nanodrop Technologies). We isolated DNA from lung tissue samples by the same procedure, except that the duration of each bead beating step was increased to 2 minutes.

### 16S Quantitative PCR

qPCR was used to quantify the 16S content of our samples. Reactions were performed on a Lightcycler 480 (Roche) using the following protocol: 50°C for 2 min, 95°C for 10 min, followed by 45 cycles of 95°C for 15 sec, and 60°C for 60 sec. Readings were taken in single acquisition mode. The primers and probes consisted of a forward-primer TCC TAC GGG AGG CAG CAG T, the reverse-primer GGA CTA CCA GGG TAT CTA ATC TT, and the 16S specific probe 5′-FAM/CGT ATT ACC GCG GCT GCT GGC AC/3′-TAMSp. A standard curve was constructed using 24 two-fold dilutions of *Helicobacter hepaticus* DNA (a bacterium known to have only a single copy of the 16S gene in its genome) beginning at 1000 ng. Samples were run in duplicate and at 1∶10 and 1∶100 fold dilutions.

### 454 Pyrosequencing

The bacterial tag-encoded FLX-Titanium amplicon pyrosequencing (bTEFAP) method targeting the V1-V3 variable regions of 16S rRNA was used to create amplicon libraries [20]. V1-V3 primer sets corresponded to 27F (5′- GAGTTTGATCNTGGCTCAG-3′) and 519R (5′- GWNTTACNGCGGCKGCTG-3′), along with appropriate sample nucleotide bar codes and the Roche A & B primers. The pyrosequencing was performed following established protocols [21].

### Data Analysis

#### Taxonomy

A locally run version of RDP Classifier (http://rdp.cme.msu.edu) was used for phylotyping 16S rDNA sequences. Sequences containing fewer than 50 nucleotides, and sequences without a valid barcode or those that had the barcode in the wrong position, were removed as low-quality reads. A confidence cut-off of 50% was used to produce accurate taxonomic identifications [22]. Data tables were constructed from the Classifier output and analyzed using several custom R scripts and the vegan package for R (http://CRAN.R-project.org/package=vegan) [23].

#### Operational Taxonomic Units (OTUs)

The open-source, platform-independent, community-supported software program, mothur (http://www.mothur.org; [24]), was used to process and analyze the sequence data. Sequence reads were cleaned and filtered using quality control procedures described above, pre-clustering, and chimera elimination. 16S rDNA analysis was performed using an OTU cutoff of 3% and followed the Costello Stool Analysis example (http://www.mothur.org/wiki/Costello_stool_analysis).

### Statistical analysis

Statistical analyses were performed using Prism 5 (GraphPad Software) for One-way ANOVA and R (http://www.r-project.org).

## Results

The characteristics of the 14 patients undergoing BAL are presented in Table 1. The age ranged from 40-78 yrs (median 53.9 yrs) with 7/14 male and 8/14 currently smoking. The six patients who underwent lung transplantation for advanced COPD were males with severe airflow obstruction (**Table 2**). One had emphysema related to alpha-1 antitrypsin deficiency (CS #6).

**Table 1:** Bronchoalveolar Lavage Patient Cohort.

Group	Subject #	Age	Ethnicity	Gender	Smoking history	FEV_1_ (%pred)	FEV_1_/FVC	Medications	Approach	Current Smoker
HS	1	53	C^1^	F	20	98	0.77	N	Oral	Yes
	2	45	C^1^	F	16	103	0.80	N	Nasal	Yes
	3	45	C^1^	M	20	114	0.93	N	Nasal	Yes
	4	49	AI/NA^2^	F	40	102	0.76	N	Nasal	Yes
	5	50	AA^3^	M	15	99	0.77	N	Nasal	Yes
	6	47	C^1^	F	39	96	0.76	N	Nasal	Yes
	7	66	C^1^	M	32	110	0.80	N	Nasal	No
CS	1	54	C^1^	M	120	79	0.63	ICS^4^/LAB^5^	Nasal	No
	2	62	C^1^	M	68	78	0.68	N	Nasal	Yes
	3	40	AA^3^	M	25	79	0.67	N	Oral	Yes
	4	60	C^1^	M	41	25	0.41	ICS^4^/LAB^5^	Oral	No
NS	1	48	C^1^	F	0	105	0.86	N	Nasal	No
	2	78	C^1^	F	0	83	0.77	N	Nasal	No
	3	58	C^1^	F	0	142	0.80	N	Nasal	No

1 = Caucasian;

2 = American Indian/Native American;

3 = African American;

4 = Inhaled Corticosteroids;

5 = Long Acting Beta-Agonists.

**Table 2:** Explant Cohort (CS).

Subject #	Age	Ethnicity	Gender	Smoking history	FEV_1_ (%pred)	FEV_1_/FVC	Medcations
5 (SLT^6^)	66	C^1^	M	No (>6 Months)	18	0.22	ICS^4^/LAB^5^
6 (BLT^7^)	57	C^1^	M	No (>6 Months)	13	0.17	ICS^4^/LAB^5^
7 (BLT)	62	C^1^	M	No (>6 Months)	15	19	ICS
8 (SLT)	59	C^1^	M	No (>6 Months)	9	16	None
9 (SLT)	59	C^1^	M	No (>6 Months)	25	44	ICS/LAB
10 (SLT)	64	C^1^	M	No (>6 Months)	16	33	ICS/LAB

6 = Single Lung Transplant;

7 = Bilateral Lung Transplant.

**Figure 1:**
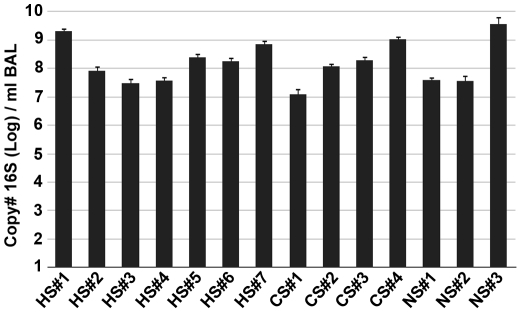
16S qPCR of BAL Samples. The number of copies of bacterial 16S per ml of BAL fluid was measured by qPCR (as described in Methods and Materials). The samples were divided into three groups: healthy smoker (HS), COPD subject (CS), and never-smoker (NS); the individual samples are displayed along the x-axis (Mean ± SEM). Samples where run in duplicate with two 10-fold dilutions.

To address our first objective of determining if there was a difference in total bacterial numbers between the three groups, we isolated total DNA from the BAL pellet after high speed centrifugation and determined 16S gene copy number by qPCR. In every sample in our study, significant levels of bacterial 16S gene signal were detected (**Figure 1**). There were no significant differences between the three study groups (Log 16S copy #/ml BAL: HS, 8.25±0.25; CS, 8.12±0.40; NS 8.24±0.66 mean ± SEM; p>0.05). Altogether, the levels of bacteria detected in the BAL fluid of our 14 subjects were consistent with previous estimates based on sterile brushings of the airways [12]. Thus, there were significant levels of bacteria in all subjects, without significant differences between never-smokers and those with end-stage lung disease.

**Figure 2:**
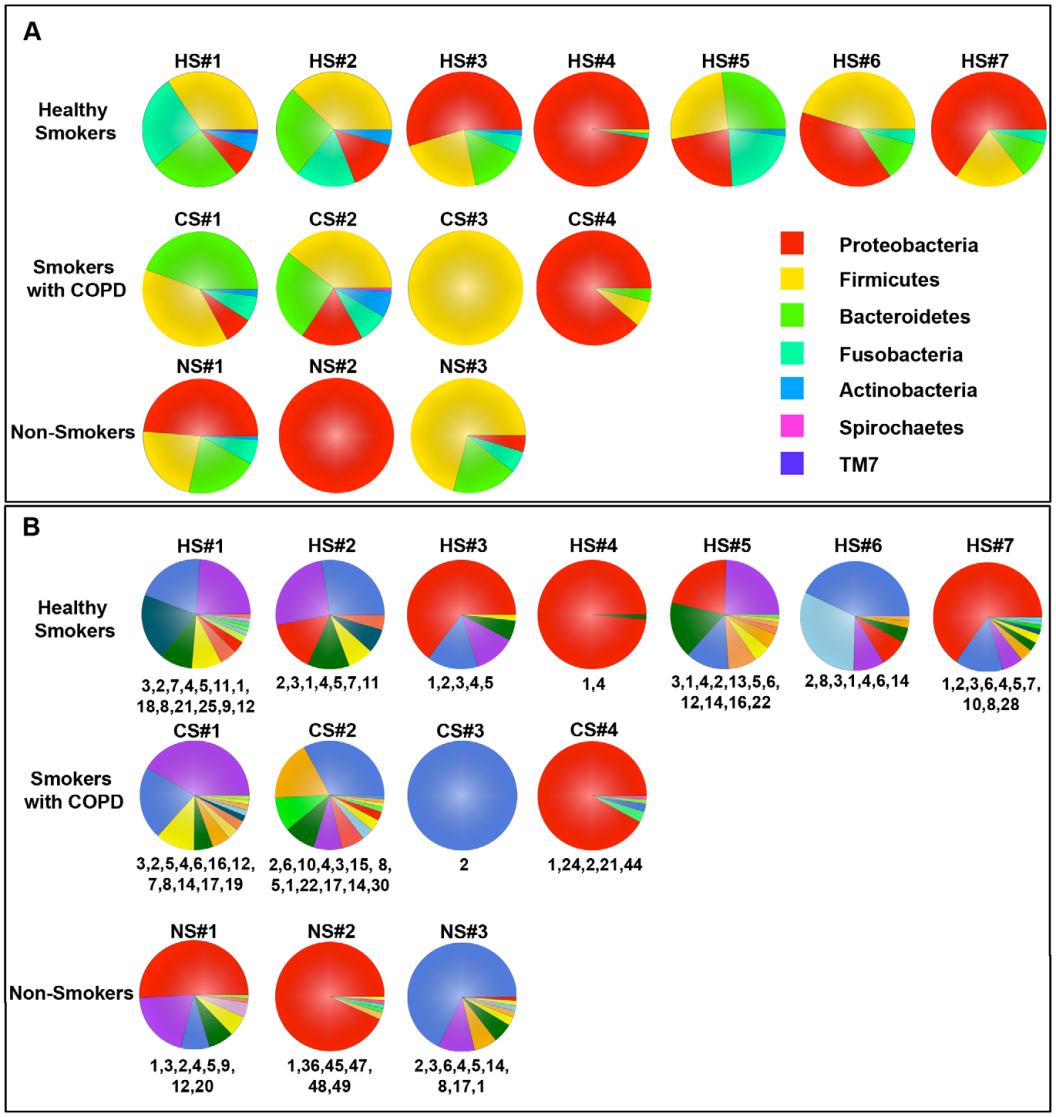
Taxonomic Classification of Bacterial Communities Present in the BAL. The V1-V3 region of the bacterial 16S genes were sequenced using 454-pyrosequencing and taxonomically classified using RDP Classifier. A. Phylum level classification of the 16S amplicons present in a given subject. B. Genus level classification of these amplicons. The numbers at the bottom of each pie chart identify the organisms which can be found in Table 3.

To compare the bacterial community structure (membership and diversity) of the resident pulmonary microbiome between subjects and between groups, we next used 454-pyrosequencing to analyze 16S amplicon libraries generated from our BAL samples. Following quality control filtering of the sequences we used RDP-Classifier [25] to assign taxonomic classifications to the sequences for ecological analysis. In agreement with our qPCR analysis (**Figure 1**), a community of lung-resident bacteria was readily identifiable in each BAL sample **(** **Figure 2** **&** **Table 3**). Virtually all of the filtered reads (91%±1.5% mean ±SEM) could be classified down to the genus level, irrespective of subject cohort. The dominant phyla in the lungs of our subjects were the Proteobacteria, Firmicutes, and Bacteroidetes (**Figure 2A**). At the phylum-level, there was heterogeneity in the bacterial communities between most of the HS group that was similar to that seen in healthy never-smokers (NS) and our two mild COPD patients (CS#1 & CS#2). By contrast, the moderate and severe COPD patients (CS#3 & CS#4) lacked bacterial community diversity, which was also noted in two healthy subjects (HS#4 and HS#7) and one never-smoker (NS#2).

**Table 3:** BAL Abundance Table.

Rank	Name	Total # Sequences	# Subjects Occurred/Total
1	Pseudomonas	78319	12/14
2	Streptococcus	23253	12/14
3	Prevotella	19916	10/14
4	Fusobacterium	8784	11/14
5	Veillonella	5937	9/14
6	Porphyromonas	4366	8/14
7	Leptotrichia	3801	5/14
8	Haemophilus	2765	8/14
9	Oribacterium	1577	6/14
10	Actinobacillus	1539	4/14
11	Actinomyces	1188	6/14
12	Megasphaera	1017	4/14
13	Sneathia	879	2/14
14	Gemella	828	7/14
15	Tropheryma	783	1/14
16	Neisseria	748	4/14
17	Granulicatella	731	5/14
18	Campylobacter	535	2/14
19	Atopobium	511	3/14
20	Bulleidia	480	4/14
21	Lachnospira	474	3/14
22	Parvimonas	379	3/14
23	Flavimonas	352	3/14
24	Bacteroides	304	2/14
25	Tannerella	262	2/14
26	Hallella	210	3/14
27	Catonella	197	2/14
28	Stenotrophomonas	193	1/14
29	Selenomonas	155	1/14
30	Mycoplasma	130	1/14
31	Peptostreptococcus	124	1/14
32	Aggregatibacter	110	1/14
33	Staphylococcus	108	1/14
34	Cloacibacterium	106	1/14
35	Citrobacter	94	1/14
36	Acidovorax	93	1/14
37	Rothia	93	1/14
38	Flavobacterium	91	1/14
39	Xanthomonas	88	1/14
40	Moryella	82	1/14
41	Anaerococcus	77	1/14
42	Corynebacterium	65	1/14
43	Centipeda	62	1/14
44	Faecalibacterium	62	1/14
45	Acinetobacter	59	1/14
46	Cryobacterium	55	1/14
47	Sphingopyxis	53	1/14
48	Burkholderia	52	1/14
49	Brevundimonas	51	1/14
50	Rhodobacter	51	1/14
51	Treponema	51	1/14

At the genus-level (**Figure 2B**), the bacterial communities in the healthy smokers (HS) and never-smokers were fairly diverse with the exception of one individual in each of the groups (HS#4 and NS#2, respectively). For each phyla present in a sample, there were typically one to two dominant genera (e.g., *Pseudomonas*, *Streptococcus*, *Prevotella*, *Fusobacterium* or *Veillonella*; **Figure 2B** and **Table 3**). Similar diversity was also seen in the two mild COPD patients (CS#1 & CS#2), but a loss of diversity was observed in the COPD subjects (CS#3 & CS#4) with more severe disease. Overall, the pulmonary microbiome in our subjects was diverse, but more limited than is typically found for bacterial communities in the mouth and intestine [26], [27].

**Figure 3:**
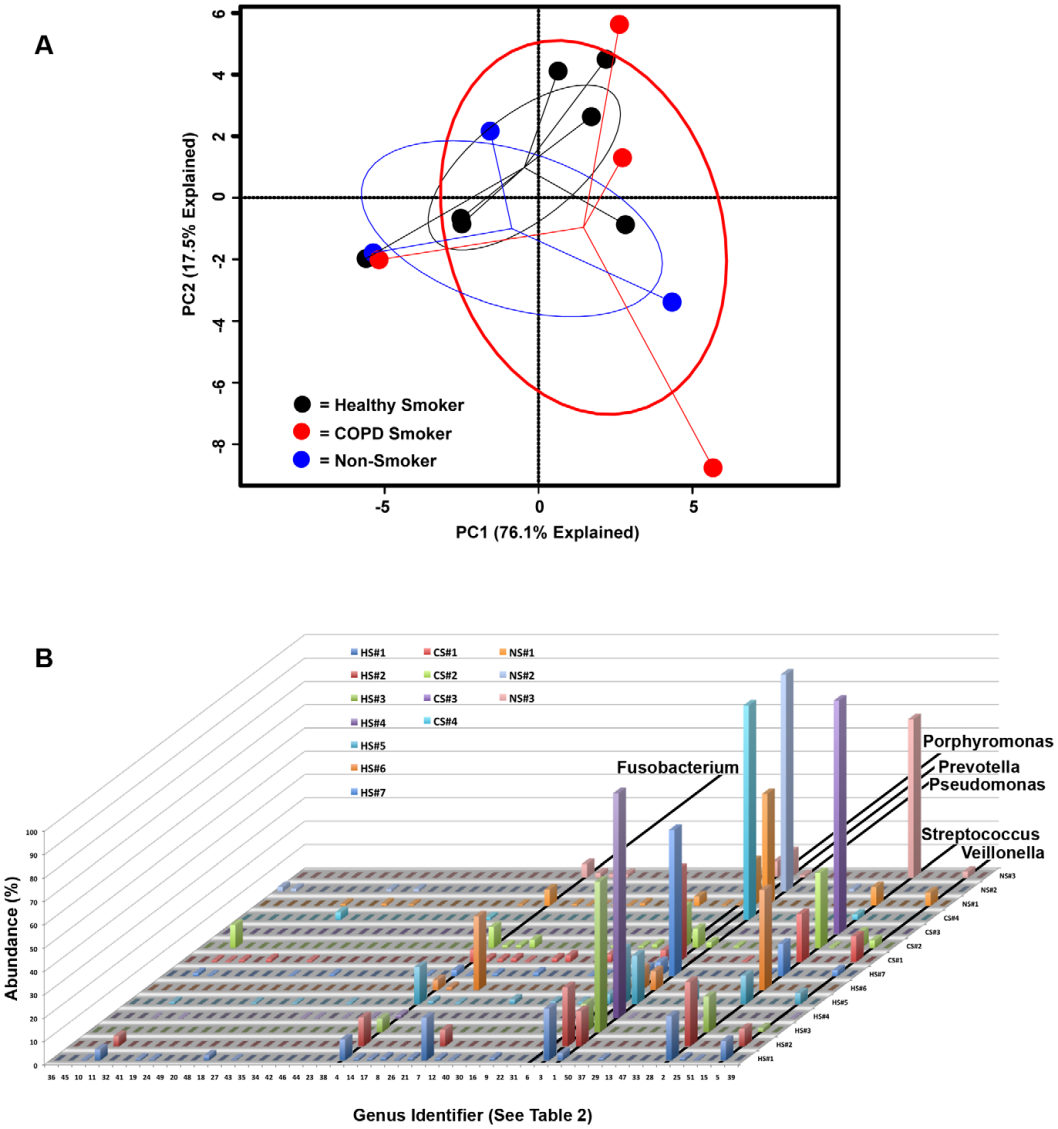
Identification of Bacterial Community Membership Overlap in Subject BALs. The relative abundance of each genera present in the BAL of each subject are plotted together with the numbers along the x-axis corresponding to the rank from Table 3. The study subject is displayed along the z-axis and the relative abundance (as a percent) is displayed along the y-axis. The genera *Pseudomonas*, *Streptococcus*, *Prevotella*, *Fusobacterium*, *Veillonella and Prophyromonas* are highlighted due to the dominance of these organisms within all of the subjects examined.

Next we analyzed the genus-level bacterial community data using principal components analysis (PCA, **Figure 3A**) to create a community ordination and examine which elements of the community have the strongest influence in the variation between subjects. This approach revealed extensive overlap in membership between the bacterial communities of the HS, CS, and NS groups. There were no bacteria that were common within a group but instead unique across groups that would separate one group from another. Thus, outgrowth from within the community, rather than invasion, seems plausible in subjects whose pulmonary microbiome was dominated by a single bacterial genus.

Our data also suggested that there may be bacteria that comprise a “core” pulmonary microbiome, i.e., found with very high frequency (at >1% of all 16 s reads) in the BAL of healthy subjects. Candidate genera that were found in greater than 75% of our subjects included *Pseudomonas*, *Streptococcus*, *Prevotella and Fusobacterium* (**Table 3** & **Figure 3B**). *Haemophilus*, *Veillonella*, *and Porphyromonas* were also identified in over half of the samples.

We also analyzed the 16S pyrosequencing data by the complementary approach of self-assembling operational taxonomic unit (OTU) analysis, which eliminates any potential binning biases inherent in taxonomic methods. For a point of reference, a 3% difference between two full-length 16S sequences is roughly equivalent to a species level difference at the genomic level [28]. We used this level of similarity to generate OTUs and calculated diversity indices using the non-parametric form of the Shannon Diversity index. Consistent with the taxonomic analysis (**Figure 2B**), OTU-based analysis (**Table 4**) confirmed that there were diverse bacterial communities (higher np Shannon values indicate higher diversity) in the healthy smokers (HS), which was similar to that seen in the healthy never-smokers (NS) and our two mild COPD patients (CS#1 & CS#2). This analysis again identified that the pulmonary microbiome was much less diverse in the moderate and severe COPD patients (CS#3 & CS#4) (**Table 4**).

**Table 4:** BAL OTU Data.

Subject #	OTUs^1^>1% of Population	Diversity (np Shannon)	OTUs^1^>1%/Genera>1%
HS#1	25	4.85	25/12
HS#2	17	3.98	9/9
HS#3	3	2.03	3/2
HS#4	31	4.29	31/11
HS#5	16	4.82	16/10
HS#6	15	3.40	15/7
HS#7	14	3.5	14/10
CS#1	20	4.91	20/12
CS#2	15	3.97	17/13
CS#3	10	1.97	10/1
CS#4	5	1.56	5/3
NS#1	18	3.42	18/8
NS#2	6	1.68	6/6
NS#3	12	3.67	12/9

To generate an estimate of the average species richness within the genera from a subject, we also compared the number of genera from the classifier-based method to the number of OTU at the 3% identity level. We limited our analyses to the number of genera that were present at >1% and the number of OTU present at >1%, respectively. Importantly, this analysis demonstrated that species-level diversity within the human lungs is very limited: approximately two OTU per genera in each subject, with the notable exception of CS#3 which had 10 (**Table 4**). Overall, both OTU and classifier-based approaches demonstrated that the lungs of all subjects contain a diverse resident bacterial microbiome that displays only limited richness at the sub-genus level.

**Figure 4:**
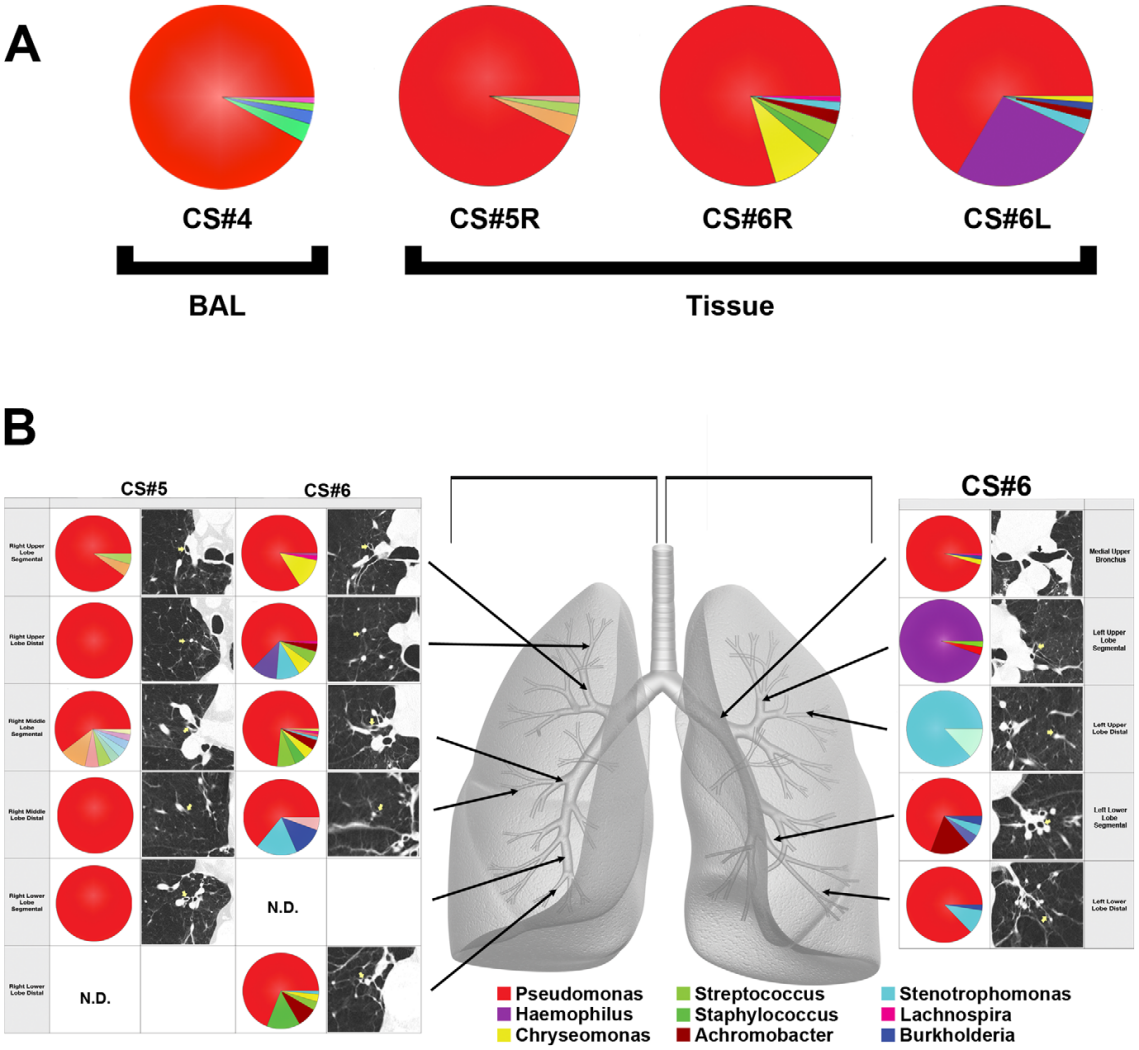
Bacterial Communities Present in Individual Lung Airways. A. Bacterial community profiles for an entire explanted lung lobe from subjects with severe COPD. The total aggregate genus level reads of samples taken from the right lung of subject CS#5, the right lung of subject CS#6, and the left lung of subject CS#6 were analyzed on a per lung basis and compared to the BAL of subject CS#4 (reproduced from Figure 2). B. Multiple samples were taken from lung explants (right lung, subject CS#5; both lungs, subject CS#6) at the time of elective transplantation. Samples were harvested from the regions of lung indicated by the arrows on the gray lung schematic. Pie diagrams depict the genus level classification of 16S sequences, and the CT images demonstrate the absence of bronchiectasis in the airways adjacent to where samples were obtained. The key for the nine most abundant organisms is provided below the lung schematic. The full community breakdown for each of the airways can be found in Table S1.

To address the critical question of whether the bacteria in the BAL samples might reflect upper airway contamination of the bronchoscopes used during the procedure, we sampled multiple tissue sites from eight COPD lung explants removed during transplantation (six single and two bilateral transplants). All tissues sampled from the explanted lungs contained readily identifiable bacterial communities (**Figure 4**). Because BAL samples multiple airways and alveoli distal to a segmental or subsegmental bronchus, we first combined all of the individual sequencing reads *in silico* from all of the tissue samples of single lobe of the surgical specimens, and performed a separate analysis. The bacterial community profile of each of the three lobes were dominated in all three samples by the genus *Pseudomonas* (**Figure 4A** **, Tissue**), which was very similar to that of the BAL sample from our severe COPD subject (CS#4) and a number of others (**Figure 2**). Thus, direct sampling of explant tissue demonstrated that the bacterial communities in the BAL samples were lung-resident microbes and not the result of bronchoscope contamination.

**Figure 5:**
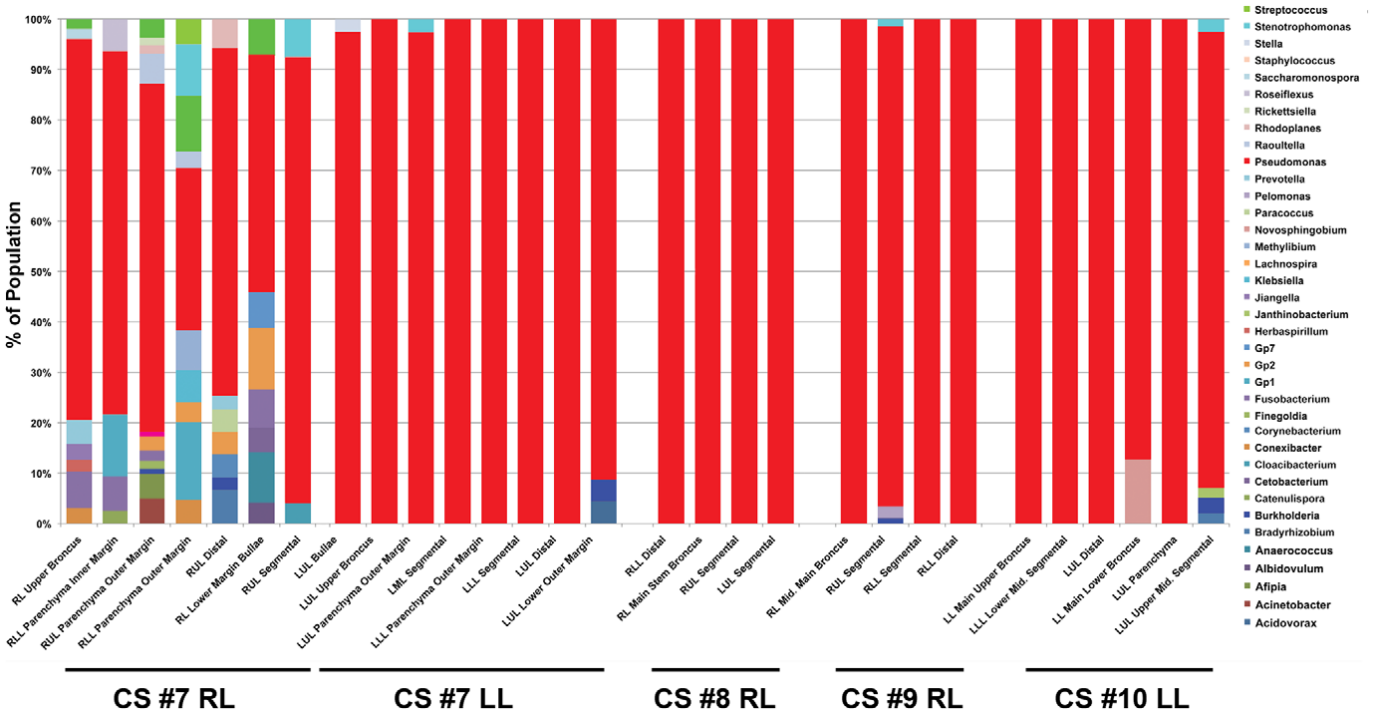
Bacterial Distribution Throughout COPD Lung Explants. Bacterial communities were characterized in the airways from 5 lobes of 4 lung explants. Multiple samples (4-8) were taken throughout the lung explants at the time of elective transplantation. The barchart depicts the genus level classification of 16S sequences identified.

We next compared individual tissue sites to determine whether there were micro-anatomic differences in bacterial communities within the lungs of subjects with advanced COPD. Each lobe had four to eight distinct tissue sites sampled, for a total of 44 tissue sites. No bronchiectatic changes were evident in the pre-operative CT images for any of the tissue sites sampled in these subjects. Figure 4b demonstrates illustrative CT images of two patients; similar results were seen in the other four patients (data not shown). However, despite normal structure, there were sites of significant differences in bacterial community composition within the same lung. This was particularly evident in the left upper lobe from subject CS#6. *Haemophilus* dominated the community in the segmental bronchus of the LUL. *Stentrophomonas* dominated in the distal bronchus of the LUL. In sharp contrast, *Pseudomonas* dominated the community in the middle upper bronchus and many others within the same lung. The additional explanted lobes also displayed micro-anatomic heterogeneity. The most striking differences were observed between tissue sites in CS#7RL, while all the tissue sites in CS#8RL were dominated almost entirely by *Pseudomonas* (**Figure 5**). These data demonstrate for the first time that marked regional differences in the bacterial microbiome can exist within an individual subject.

**Figure 6:**
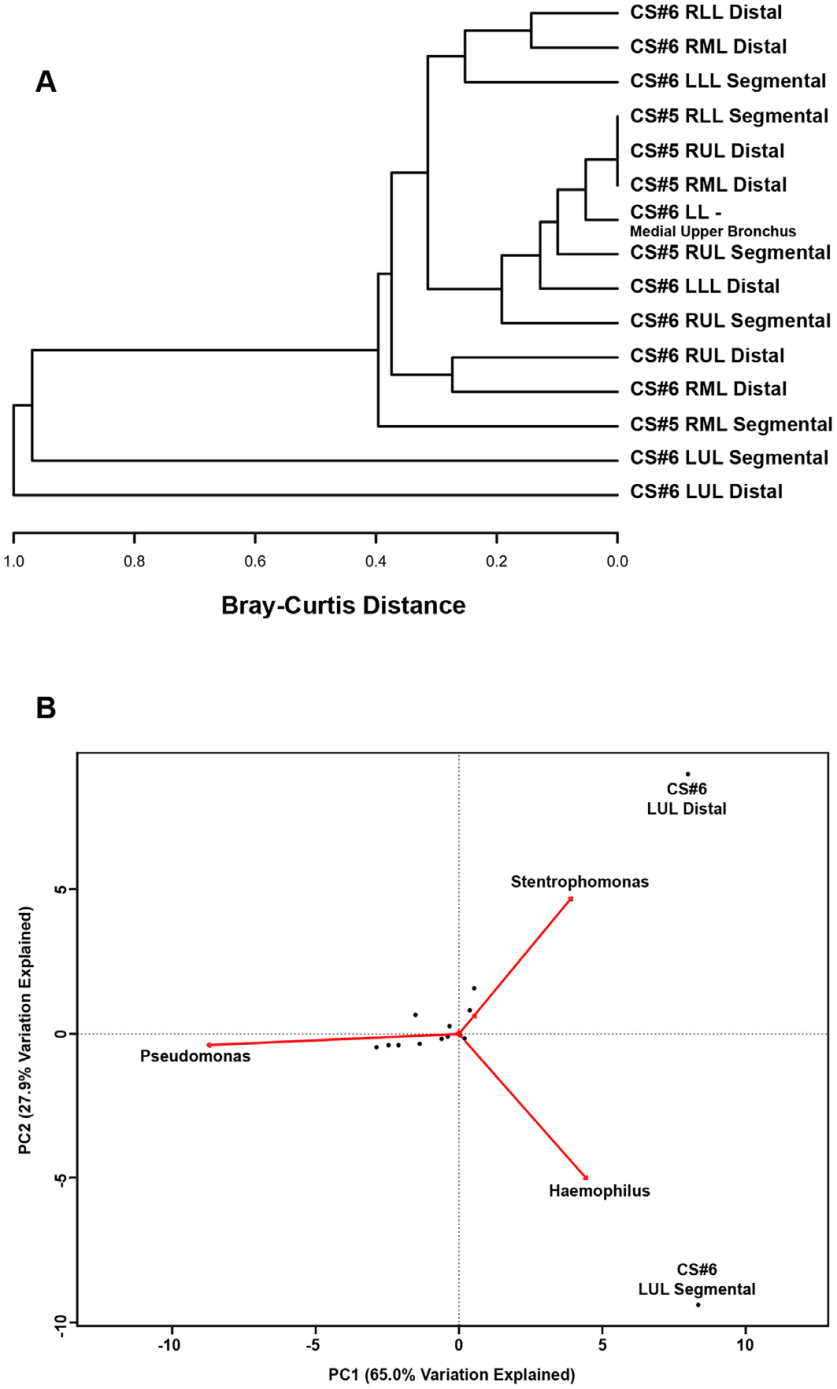
Cluster Analysis of the Bacterial Communities Sampled from Sites. A. Furthest-neighbor joining tree built on the Bray-Curtis distance and B. Biplot of the principle components analysis of the normalized bacterial communities from multiple anatomic sites in the lung explants.

We next constructed a furthest-neighbor joining tree, based on the Bray-Curtis distance, of sampled communities to compare the beta-diversity (inter-community diversity) between the bacterial communities of the different sites from CS#5 and CS#6 (**Figure 6A**). Most of the samples resulted in a low Bray-Curtis distance, indicating that the samples are more similar to each other. However, the CS#6 LUL Segmental and CS#6 LUL Distal samples cluster apart from the main group and from each other with high Bray-Curtis values, indicating a high degree of dissimilarity. We again used PCA ordination to identify which elements were responsible for driving the differences between individual samples (**Figure 6B**). This analysis demonstrated that the micro-anatomic variation in the samples was driven by either the dominance of *Pseudomonas*, *Haemophilus*, or *Sentrophomonas* at the site.

Analysis of OTUs (based on 3% dissimilarity) demonstrated that significant numeric differences also existed between the number of OTUs in tissue sites within the same lung and even within the same lobe (**Table 5**). One intriguing observation was that in samples in which there was domination by a single genus (*Pseudomonas*), there was often marked heterogeneity in the numbers of OTUs. For example, we observed this for a number of regions in the right lobe from CS#5 and in the BAL from subject CS#3 (**Figure 2B** **&** **Table 3**). The OTU and classifier-based analyses of these samples were consistent with each other and clearly demonstrated that significant micro-anatomic differences can exist in bacterial communities within the same lung of subjects with advanced COPD.

**Table 5:** Explant OTU Data.

Subject #	Location	OTUs>1% of Population	Diversity (np-Shannon)	OTUs^1^>1%/Genera>1%
CS5	RUL Segmental	7	1.85	7/3
	RUL Distal	4	1.11	4/1
	RML Segmental	19	2.86	19/9
	RML Distal	5	1.59	5/1
	RLL Segmental	5	1.30	5/1
CS6	RUL Segmental	4	1.57	4/4
	RUL Distal	11	2.48	11/7
	RML Segmental	14	2.57	14/9
	RML Distal	4	1.76	4/4
	RLL Distal	11	2.61	11/6
	Medial Upper Bronchus	5	1.26	5/4
	LUL Segmental	9	1.73	9/3
	LUL Distal	7	1.59	7/2
	LLL Segmental	7	1.99	7/5

## Discussion

This study using massively parallel pyrosequencing of bacterial 16S amplicons provides novel information on the microbiota of a range of subjects including healthy non-smokers, smokers with normal lung function, and stable COPD subjects with mild or severe spirometric disease. We have demonstrated three key findings. First, the lungs of healthy smokers contain a bacterial microbiome that is quantitatively significant, diverse (but of limited membership), and quite distinct from that reported for the oral cavity or nasopharynx [26]. Second, the diversity of the lung bacterial microbiome is often lower in subjects with decreased lung function, most commonly associated with dominance by *Pseudomonas spp*. Third, this is the first study to describe that the numerous microanatomic sites within the lung can give rise to significant differences in bacterial community structure.

Our current study has examined the lung microbiome with an unprecedented depth, averaging ∼12,000 sequences per sample. This markedly greater sequencing depth increases confidence that we have sufficiently sampled the lungs to characterize the microbial lung community accurately. Our studies of the BAL from healthy smokers are consistent with the recent demonstration of a diverse bacterial lung microbiome in healthy individuals [12], a study which had ∼3,000 sequence reads total for the entire study.

Importantly, our finding that some smokers had a less diverse lung microbiota relative to smokers with normal lung function indicates that alteration in lung microbiota can occur in subjects with no spirometric evidence of disease. Whether this relative reduction in diversity is persistent, is an effect of the inflammatory changes that characterize COPD, or could in part contribute to disease progression are all questions that will require longitudinal follow-up in larger groups of subjects. Our results are consistent with findings at another mucosal site, the gastrointestinal tract, where decreases in the diversity of the microbiota are associated with increased incidence of inflammatory bowel disease [30], [31]. Lung community dysbiosis could provide the constant inflammatory stimulus that has long been observed in COPD [32]. Thus, in the lungs, as with other sites on the mucosa, a diverse microbiota may be important for health, including colonization resistance, epithelial integrity, and immunoregulation [33], [34].

Collectively, our results provide a unifying framework for characterizing the role of *Pseudomonas* and *Haemophilus* in the development, progression and/or exacerbation of COPD. Prior to the use of culture-independent techniques, *Haemophilus* was the organism most frequently grown from samples of COPD lungs [35], with *Pseudomonas* oftentimes noted [36]. The current work suggests that these organisms are generally present even under healthy conditions. While larger studies must be performed, our data support a model where dominance of an organism within the lung's microbial community is associated with disease.

We have also shown that because of local differences in lung airway microarchitecture, samples from different airways taken throughout the lungs can contain very different bacterial communities. This result was most pronounced in the airways of the left upper lobe in one transplant recipient (**Figure 4**) where the bacterial community was dominated by a bacterial genera (*Haemophilus*) that was not detected at high levels in the other airways. A previous study [12] found a significant correlation between COPD and the presence of *Haemophilus* spp. in sterile brushings of the left upper lobes. At first pass, this finding appears to conflict with the domination by *Pseudomonas* spp. of BAL samples in our study and of endotracheal aspirates in a study of severe acute exacerbations of COPD [13]. However, since COPD pathology may be anatomically heterogeneous, our observation that the lung microarchitecture allows for the development of distinct localized microbial communities during disease provides a unifying hypothesis for all the culture-based and -independent studies on the role of bacteria in COPD progression.

The demonstration of spatially distinct bacterial communities in the lungs may be very important to our understanding of pulmonary health and disease because anatomic variation in the microbiome is becoming the focus for dissecting disease mechanisms at other body sites. For example, regions of skin prone to dermatitis have been shown to have different microbial communities when compared to adjacent areas that remain disease-free [37]. Similarly, differences between teeth in the structure of the microbial community predisposes to disease within the same mouth [38]. Mechanistically, the microbial communities in teeth with periodontal disease are locally enriched for methanogenic Archea when compared to those that are disease-free [38]. Although these organisms are not disease-causing, the evidence suggests that they alter the local environment such that bacteria known to cause disease can thrive. It is possible that similar pathogenic syntropic interactions also exist in the lungs. We observed the most striking microanatomic community differences in the upper lobes of the lung, which is consistent with clinical observations that emphysema in COPD most commonly begins in the upper lobes.

Recognized limitations of our study include the absence of oropharyngeal samples and the relatively small sample size. While the latter is a matter for future studies, the former will be dealt with here. The oropharyngeal sample as a control for microbiologic studies of the lung is rooted in the belief that the lungs should be sterile; thus, the purpose of the oropharyngeal sample is to rule out contamination. However, despite the lack of this sample, we can demonstrate that the lung microbiota that we have identified is not the result of contamination. First, the levels of 16S detected in subject BAL are too high to be consistent with contamination (Figure 1), but high enough to be consistent with a low level colonization. Second, the domination of Proteobacteria, in particular *Pseudomonas spp.*, in BAL samples is radically different from the nasal cavity, which is dominated by the phylum Actinobacteria [26], and the oropharynx, which is dominated by Firmicutes [26]. In some subjects, Proteobacteria have been shown to have a larger presence in the oropharynx, but Pseudomonads were never encountered [26]. Finally, when the airways from explanted lungs were sampled directly, the bacteria identified were the same as those identified in BAL samples. However, all of our advanced COPD explants lacked the diversity observed in the “healthy smoker” controls. Collectively, these data argue strongly that the airways of the lungs are an independent microbial habitat, and that our results are not simply a result of contamination.

In summary, these results demonstrate the need to consider, in a systematic, anatomically-correlated fashion, both the lung microbiome and the host inflammatory response when studying COPD. We believe that CT imaging will be central to this endeavor. By demonstrating that one person's lungs can harbor both generalized areas of “healthy” microbiome and a single site containing a “pathogenic” community, our results suggest a mechanism by which the interaction of lung pathogens and host immunity might contribute to localized disease progression, even in the absence of overt exacerbation.

## Supporting Information

Table S1 Table S1 depicts the complete population breakdown of the bacterial genera present in the lung explant tissue samples shown in Figure 4.
(DOC)
