# Supplementary material for: Analysis of the Lung Microbiome in the “Healthy” Smoker and in COPD
Source: PLoS One. 2011 Feb 22;6(2):e16384. doi: 10.1371/journal.pone.0016384 (PMC3043049; doi:10.1371/journal.pone.0016384)
Supplement: Table S1 — depicts the complete population breakdown of the bacterial genera present in the lung explant tissue samples shown in Figure 4. (DOC) [file pone.0016384.s001.doc]

Supplemental Table S1

| **Sample** | **Location** | **Genera Present** |
| --- | --- | --- |
| **CS#5** | **Pooled RL Total:** | Pseudomonas (89%); Acidovorax (4%); Acinetobacter (2%); Allobaculum (1%); Others (5 genera contributing < 1% to population each) |
|  | **RUL Segmental** | Pseudomonas (90%); Acidovorax (6%); Acinetobacter (4%) |
|  | **RUL Distal** | Pseudomonas (100%) |
|  | **RML Segmental** | Pseudomonas (60%); Acidovorax (11%); Allobaculum (6%); Acinetobacter (6%); Sphingopyxis (4%); Brevundimonas (4%); Serratia (4%); Gp4 (3%); Roseateles (2%) |
|  | **RML DIstal** | Pseudomonas (100%) |
|  | **RLL Segmental** | Pseudomonas (100%) |
| **CS#6** | **Pooled RL Total** | Pseudomonas (76%); Chryseomonas (9%); Staphylococcus (3%); Streptococcus (3%); Achromobacter (2%); Stenotrophomonas (2%); Lachnospira (1%); Others (23 genera contributing < 1% to population each) |
|  | **RUL Segmental** | Pseudomonas (81%); Chryseomonas (13%); Lachnospira (2%); Faecalibacterium (1%); Others (13 genera contributing < 1% to population each) |
|  | **RUL Distal** | Pseudomonas (63%); Duganella (11%); Stenotrophomonas (10%); Chryseomonas (6%); Streptococcus (5%); Achromobacter (3); Lachnospira (1%) |
|  | **RML Segmental** | Pseudomonas (72%); Streptococcus (8%); Staphylococcus (5%); Chryseomonas (5%); Achromobacter (4%); Stenotrophomonas (1%); Prevotella (1%); Shewanella (1%); Peptoniphilus (1%); Others (3 genera contributing < 1% to population each) |
|  | **RML DIstal** | Pseudomonas (64%); Stenotrophomonas (18%); Burkholderia (13%); Cloacibacterium (5%); |
|  | **RLL Distal** | Pseudomonas (69%); Staphylococcus (14%); Achromobacter (8%); Streptococcus (4%); Chryseomonas (3%); Stenotrophomonas (1%) |
|  |  |  |
|  | **Pooled LL Total** | Pseudomonas (65%); Haemophilus (26%); Stenotrophomonas (3%); Achromobacter (2%); Burkholderia (1%); Chryseomonas (1%); Others (6 genera contributing < 1% to population each) |
|  | **Medial Upper Bronchus** | Pseudomonas (95%); Chryseomonas (2%); Burkholderia (1%); Shewanella (1%) |
|  | **LUL Segmental** | Haemophilus (93%); Pseudomonas (3%); Streptococcus (2%); Others (3 genera contributing < 1% to population each) |
|  | **LUL Distal** | Stenotrophomonas (87%); Escherichia (13%) |
|  | **LLL Segmental** | Pseudomonas (69%); Achromobacter (17%); Pelomonas (5%); Stenotrophomonas (5%); Burkholderia (4%) |
|  | **LLL Distal** | Pseudomonas (87%); Stenotrophomonas (10%); Burkholderia (2%) |
